# Insights from the in silico structural, functional and phylogenetic characterization of canine lysyl oxidase protein

**DOI:** 10.1186/s43141-020-00034-w

**Published:** 2020-06-16

**Authors:** Afnan Saleem, Shiveeli Rajput

**Affiliations:** 1 Division of Animal Biotechnology, F.V.Sc & A.H, SKUAST-Kashmir, Srinagar, India; 2 Division of Animal Biotechnology, NDRI, Karnal, India

**Keywords:** In silico, Lysyl oxidase, Canine, Homology modeling, Protein

## Abstract

**Background:**

Lysyl oxidase is an extracellular regulatory enzyme with an imperative role in interlinking of collagen and elastin by oxidizing lysine residues. Lysyl oxidase has been implicated in incidence of mammary tumors in bitches. Therefore, it becomes significant to study the structural and functional features of this enzyme for a better understanding of its molecular mechanisms.

**Results:**

The detailed computational investigation of the canine lysyl oxidase protein was analyzed in silico with respect to its physicochemical properties, secondary and tertiary structure predictions and functional analysis using standard bioinformatic tools. Lysyl oxidase is a flexible protein with an average molecular weight of around 46 kDa, unstable, hydrophilic, and extracellular (secretory) in nature. Twelve cysteine residues and a disulfide bridge were also found. Secondary structure analysis shows that most of the protein has predominant coiled configuration. A putative copper-binding region signature was predicted. The phylogenetic relationship of canine lysyl oxidase with a vast range of mammalian species indicates that the protein was very well conserved throughout the course of evolution. Top 10 interacting proteins were identified using STRING v10.0 analysis, elastin being the closest interacting protein. Functional analysis by InterproScan predicted protein’s biological role in oxidation-reduction process.

**Conclusion:**

Understanding the structural and functional properties of the protein will facilitate a better understanding of its mechanism of enzyme action. Further, the predicted 3D model will serve as a cornerstone for further understanding towards the tumorigenesis potential of the protein.

## Background

Lysyl oxidase, a copper-dependent amine oxidase belongs to the oxido reductase family of enzymes and is secreted in the extracellular space [1]. LOX plays a major role in collagen and elastin crosslinking by acting on the peptidyl lysine and converting it into reactive aldehyde (allysine) which is vital for collagen fibrils stabilization and maintaining the integrity of mature elastin [2]. In a LOX knockout mice model, the physiological significance of lysyl oxidase-mediated crosslinking was illustrated due to severe fragility of the connective tissue of the cardiovascular system leading to the death of the mice either before or shortly after birth [3].

In canines, *lox* gene is mapped on chromosome 11. Canine *lox* contains 7 exons and spans across a genomic region of ~8.16 kb (from 12031440 bp to 12039603 bp) [4]. LOX fragment has three segments that is, signal peptide, propeptide, and mature LOX. By 1990s, five different LOX genes (LOX, LOXL1, LOXL2, LOXL3, and LOXL4) encoding proteins were identified which shared a highly conserved C terminal domain and a diverse N-terminal. Lysyl oxidase enzyme family contains a catalytic domain and a cytokine receptor-like domain at their C termini. Catalytic domain has a copper-binding motif and a unique lysyl-tyrosylquinone (LTQ) cofactor. The coordination of copper into the active site is brought by four histidines of copper-binding motif [5]. The LTQ cofactor is conserved in all LOX like proteins and is essential for the catalytic activity of LOX.

LOX proteins are reported not only in animals but also in archaea, bacteria, and many other eukaryotes revealing a pre-metazoan origin for the LOX family. Based on the present understanding of the mammalian LOX genes, LOX/L1/L5 and LOXL2/L3/L4 are the two LOX superfamilies. LOX/L1/L5 superfamily has been reported in cnidarians and chordates. However, LOXL2/L3/L4 superfamily is present in bilaterian genomes and is reportedly lost in cnidarians. This LOX family is present in protostomes, tunicates, and cephalochordates. Vertebrates are known to have the highest LOX enzymes with all well-known five families (LOX, LOXL1, LOXL2, LOXL3, and LOXL4), one specific family to fishes (LOXL5) and one specific to lampreys (LOXL2/L3/L4). However, no LOX gene was identified either in nematodes, ctenophore, or placozoan [6].

Lysyl oxidase genes are a family of LOX paralogs suggesting diverse functions due to the different regulation of the LOX family. Novel functions of the LOX family include collagen and elastin crosslinking, tumor progression [7], histone protein modification [8], and chemotaxis [9]. LOX activity has been demonstrated in various fibrotic diseases, connective tissue disorders, and hypoxia-induced tumors [10]. Also, over expression of LOX is considered tumor marker for invasiveness in breast cancer, head and neck squamous cell, prostatic and renal cell carcinomas [11]. We have previously reported elevated expression of LOX with incidence of mammary tumors in bitches [4]. Other than cancer, lysyl oxidase activity is also found reduced in nutritional copper deficiency and lathyrism [12] and in two X-linked recessively inherited disorders, Menkes disease, and occipital horn syndrome (OHS) [13].

Regardless of its important role, canine lysyl oxidase is not well characterized structurally or functionally. Considering the importance of this protein, the present study is formulated to analyze canine lysyl oxidase gene by assessing its phylogenetic relationships, physicochemical properties, secondary and tertiary structure prediction, motif prediction, and functional analysis.

## Methods

### Retrieval of nucleotides and protein

*Canis lupus famiilaris* lysyl oxidase nucleotide sequence was retrieved from NCBI (MH330152.1). Basic Local Alignment Search Tool (BLAST) was used to obtain similar sequences in other organisms. Multiple sequence alignment was carried out on all sequences using Molecular Evolutionary Genetic Analysis (MEGA) 6.0 version standalone software [14]. Sequence of lysyl oxidase protein was retrieved from UNIPROT (ID: J9NZK5_CANLF). This sequence was used as an input for Expert Protein Analysis System (ExPASy) which is the proteomic server of Swiss Institute of Bioinformatics (SIB) (https://www.expasy.org/) [15].

### Phylogenetic analysis

At least 57 organisms were selected (on the basis of highest similarity) to infer the evolutionary relationships with canine sequence as a reference point. The phylogenetic tree was constructed using maximum likelihood method of the Mega 6.0 package. The consistency of the inferred phylogenetic tree was evaluated with bootstrap analysis of 1000 replications.

### Primary structural analysis of lysyl oxidase

Primary structural analysis of the lysyl oxidase protein was determined using ProtParam from ExPASy. The biophysical and biochemical properties include molecular weight (Mw), isoelectric point(pI), extinction coefficients (EC-quantitative study of protein-protein and protein-ligand interactions) [16], instability index (II-stability of proteins) [17], aliphatic index (AI-relative volume of protein occupied by aliphatic side chains) [18], grand average hydropathicity (GRAVY-sum of all hydropathicity values of all amino acids divided by number of residues in a sequence) [19], half-life [20], and number of positive and negative residues.

### Secondary structure characterization

The coding sequence of canine lysyl oxidase gene was translated to protein sequence by using ExPASy translated tool (http://web.expasy.org/translate/). The amino acid sequence was subjected to secondary protein structure prediction by using (http://bioinf.cs.ucl.ac.uk/psipred/). Hydrophilicity plot, antigenic index [21], and surface probability plot (Emini) were predicted using protean tool—DNASTAR [22].

### Tertiary structure prediction

The tertiary structure prediction of lysyl oxidase protein was modeled through using ab initio approach using online available tool RaptorX (http://raptorx.uchicago.edu/) and Swiss model software (https://swissmodel.expasy.org/). The model, thus obtained, was further validated by Ramachandran’s plot using the RAMPAGE online tool (http://mordred.bioc.cam.ac.uk/~rapper/rampage.php).

### Functional analysis

CYC_REC tool was used to predict the SS-bonding of cysteine residues in protein sequence [23]. Potential phosphorylation sites of the protein was studied using NetPhos2.0 [24]. Glycosylation sites were predicted using NetNGlyc server (http://www.cbs.dtu.dk/services/NetNGlyc/) that is provided by Centre for Biological Sequence Analysis, Technical University of Denmark (CBS DTU). Location of signal peptide cleavage sites was predicted using Signal P-4.1 [25]. Psite is a protein domain database for functional annotation and description of protein sequences which. Motifs in the lysyl oxidase amino acid sequences were predicted using Psite software [26]. ProtComp 9.0 was used to identify the sub-cellular localization of protein [27]. Inter-ProScan (https://www.ebi.ac.uk/interpro/) functionally characterizes proteins by identifying protein families, domains, and functional sites [28].

### Protein-protein interaction study

STRINGv10.0 web server (http://string-db.org) was used to predict the interaction of lysyl oxidase protein with other closely allied proteins. Canine lysyl oxidase was chosen as the query sequence and a protein-protein interaction network was generated [29].

### Ethics approval and consent to participate

Not applicable.

## Results

### Retrieval of nucleotides and protein

A total of 57 lysyl oxidase sequences from different species were retrieved for phylogenetic analysis. Canine lysyl oxidase protein sequence was retrieved from UNIPROT and used for studying its physicochemical properties, functional analysis, protein interactions, secondary and tertiary structures using various computational tools and servers.

### Phylogenetic analysis

**Fig. 1:**
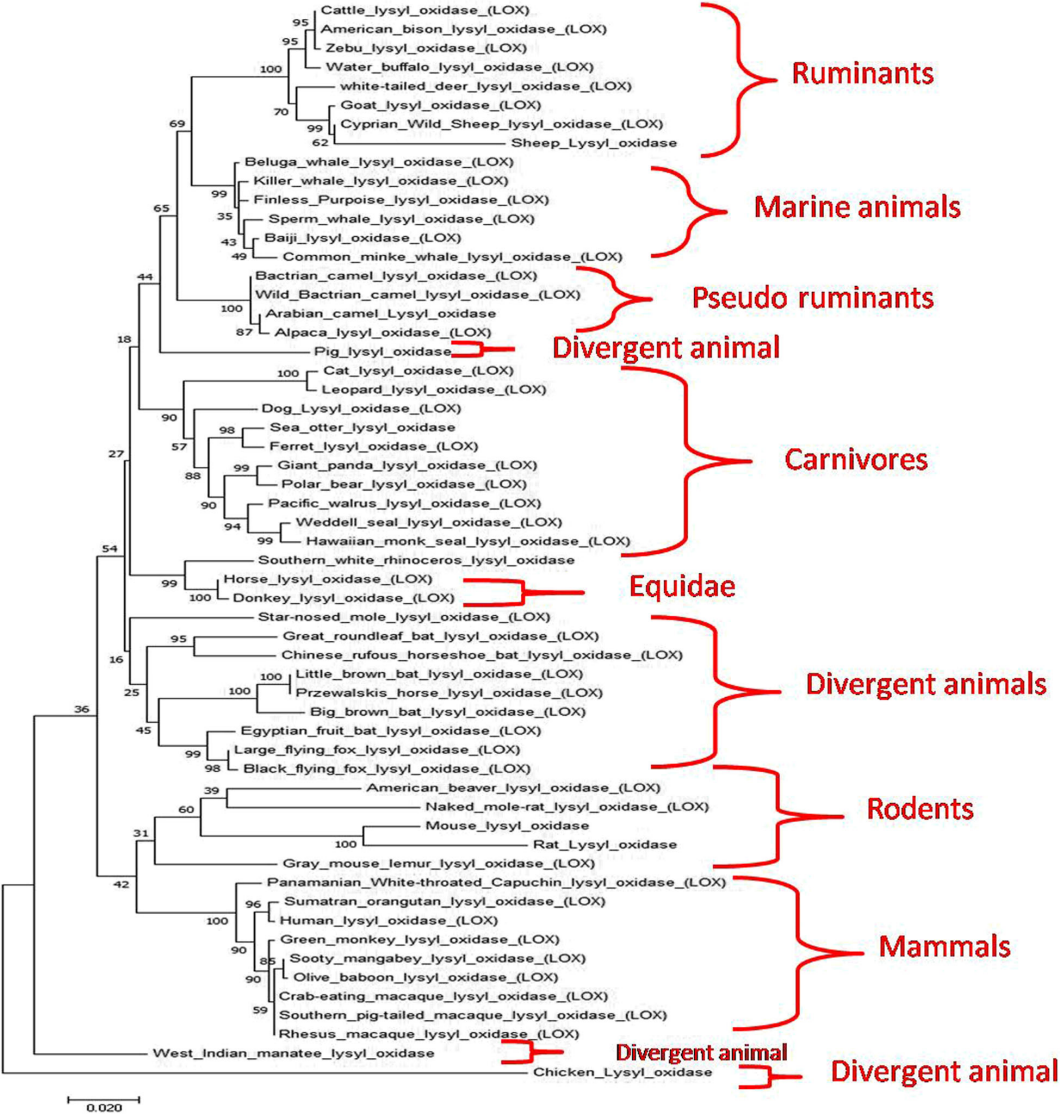
Phylogenetic tree of lysyl oxidase gene showing evolutionary relationship among different organisms using MEGA 6.0 software (Boot-straps: 1000 replicates). Canine lysyl oxidase gene showed highest immediacy with leopard while it diverged away from ruminants and camels

Phylogenetic tree depicts the formation of different clads on the basis of the evolutionary changes between sequences. Higher bootstrap values shows the higher consistency of the given data. The phylogenetic tree was constructed by subjecting 57 nucleotide sequences to maximum likelihood method (MEGA 6.0) with 1000 bootstrapping resampling. The phylogenetic tree showed that sequences belonging to the same order and family formed different clads (Fig. 1). The results showed that the gene came from the common ancestry root but diverged into different clads in the course of evolution. Ruminant lysyl oxidase (cattle, American bison, zebu, and water buffalo) are forming an independent clad and clusters away from the canine lysyl oxidase sequence. Leopard lysyl oxidase sequence was found closest to canine sequence followed by cat. The lysyl oxidase sequence of sea otter, ferret giant panda, pacific walrus, Weddell seal, and Hawaiian monk though, forming an independent clad but are quite similar to canine lysyl oxidase. Among equines, horse and donkey lysyl oxidase sequence clustered very much near to canines and are closely related. Human lysyl oxidase sequence was found to be less similar to the canine sequence. Canine lysyl oxidase sequence showed maximum divergence from camels (Arabian camel, Bactrian camel, and wild Bactrian camel). Chicken also showed divergence away from the canine sequence. To infer the evolutionary history of LOX proteins from eukaryotes, bacteria, and archaea, researchers have surveyed a wide selection of genomes in the past. A pre-metazoan source of this family has been reported so far [6].

### Primary structural analysis of lysyl oxidase

The amino acid composition (Table 1) and physicochemical properties (Table 2) of lysyl oxidase protein were assessed using ExPASy ProtParam server. The protein has 409 amino acids. Alanine is the most abundant amino acid present and proline; tyrosine are the next abundant amino acids present predominantly. The presence of aspartic acid in proteins is vital as it interacts with the solvent which further stabilizes the protein’s 3D structure.

**Table 1:** Amino acid composition of lysyl oxidase protein

Amino acid	No. of amino acid	% of amino acid
Ala (A)	45	11%
Arg (R)	39	9.5%
Asn (N)	17	4.2%
Asp(D)	25	6.1%
Cys (C)	11	2.7%
Gln (Q)	23	5.6%
Glu (E)	12	2.9%
Gly (G)	28	6.8%
His (H)	12	2.9%
Ile (I)	9	2.2%
Leu (L)	28	6.8%
Lys (K)	6	1.5%
Met (M)	5	1.2%
Phe (F)	9	2.2%
Pro (P)	37	9.0%
Ser (S)	29	7.1%
Thr (T)	19	4.6%
Trp (W)	7	1.7%
Tyr (Y)	33	8.1%
Val (V)	15	3.7%

**Table 2:** Physicochemical properties of lysyl oxidase protein

No.	Biophysical and biochemical properties	Values
1.	No. of amino acids	409
2.	Molecular weight	46,150.29
3.	Isoelectric point	8.84
4.	Negatively charged residues (Asp + Glu)	37
5.	Positively charged residues (Arg + Lys)	45
6.	Extinction coefficients	88295
7.	Abs 0.1%	1.913
8.	Instability index	50.34 (Unstable)
9.	Aliphatic index	56.92
10.	(GRAVY)	−0.757
11.	Half-life	30 h

The average molecular weight of the protein was around 46 kDa. The state of a solution where the amino acid produces the identical amount of positive and negative charges and thus, an ultimate zero charge .The isoelectric point of lysyl oxidase was found to be 8.84 which suggests that the given protein sequence seemed mildly alkaline. In isoelectric focusing method, this computed pI will be supportive for developing buffer system for purification. The instability index is computed to 50.34 which classifies the lysyl oxidase protein as unstable. The extinction coefficients was 88295 and the aliphatic index was 56.92. Relative volume of a protein occupied by it aliphatic side chains (alanine, isoleucine, leucine, and valine) is denoted by aliphatic index. The higher the aliphatic index, the higher will be the stability of the protein [30]. The grand average of hydropathy (GRAVY) value indicates the solubility of proteins and was found to be −0.757. The lesser the value is, the more superior the interaction takes place between protein with water [31]. The expected half-life was about 30 h.

### Secondary structure characterization

**Fig. 2:**
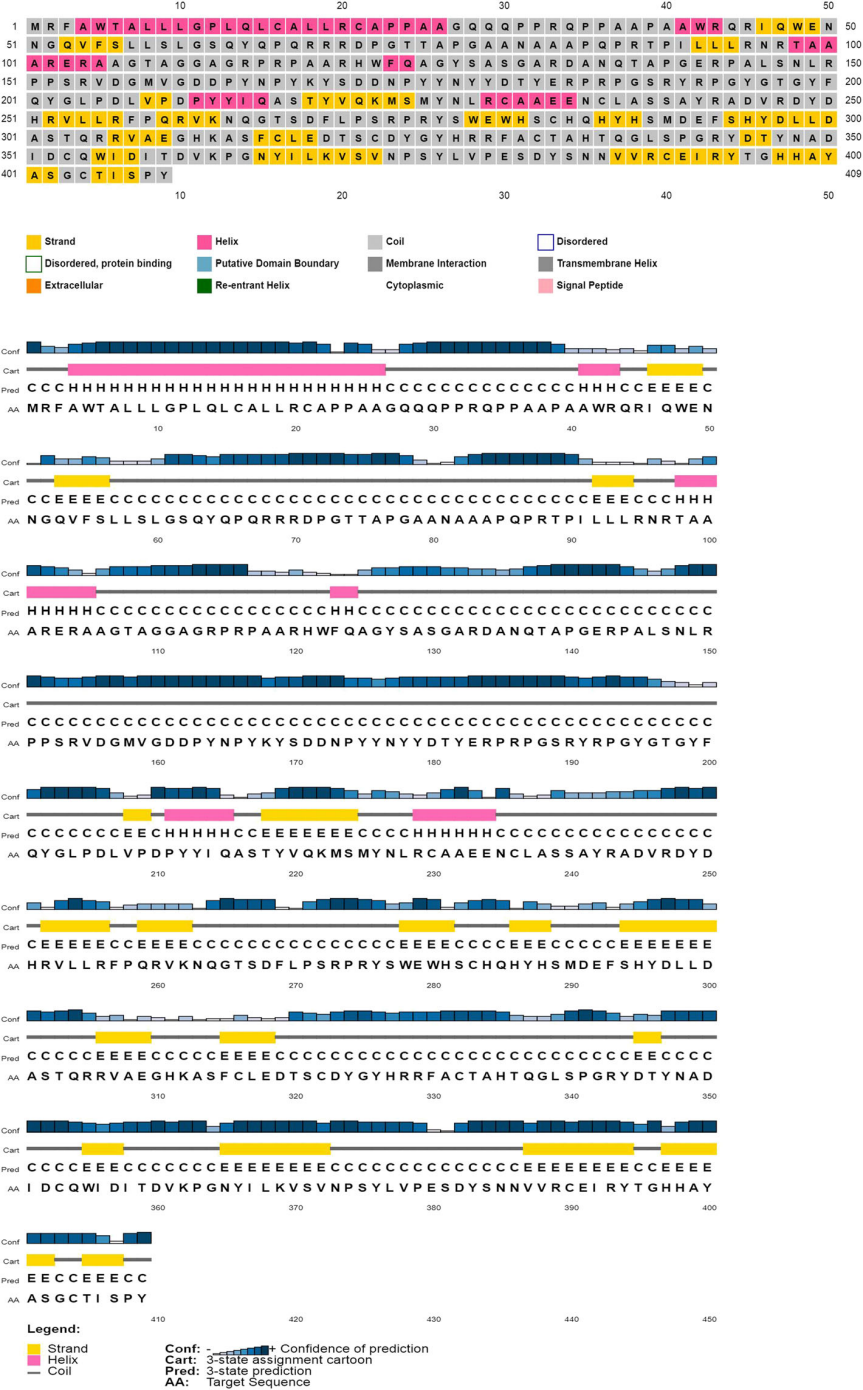
Secondary structure prediction using PSIPRED. Eight percent of total amino acids contributed to helix, 73% to coils, and 18% to strands. Thus, coil dominates the secondary structure elements followed by alpha helix

**Fig. 3:**
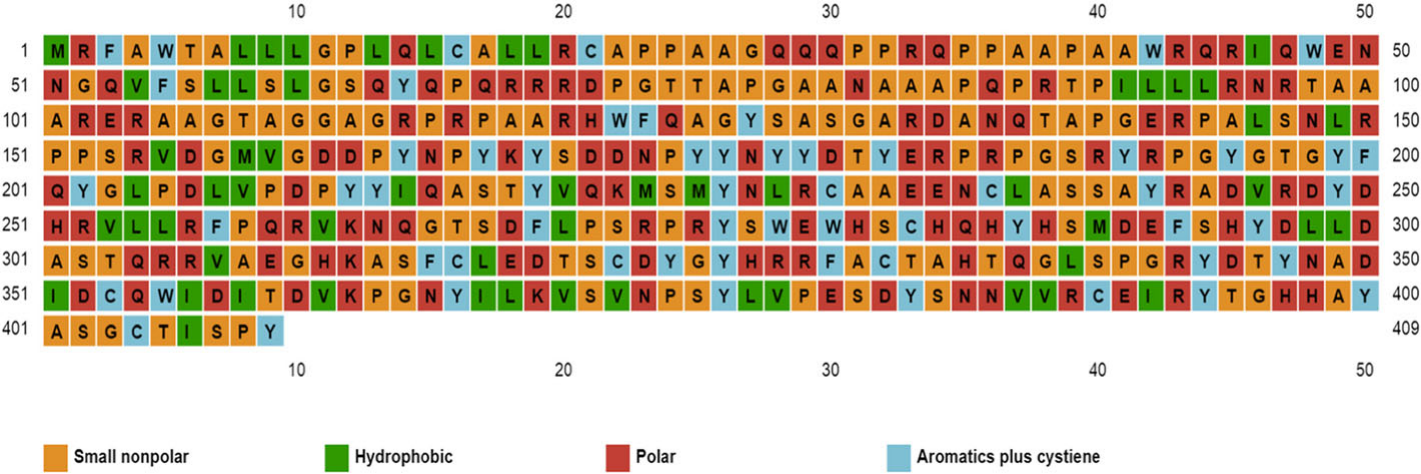
Secondary structure prediction using SWISS-MODEL. Thirty-eight percent of total amino acids were non-polar, 34% were polar, 14% were hydrophobic, and 14% contributed to aromatic plus cysteine

**Fig. 4:**
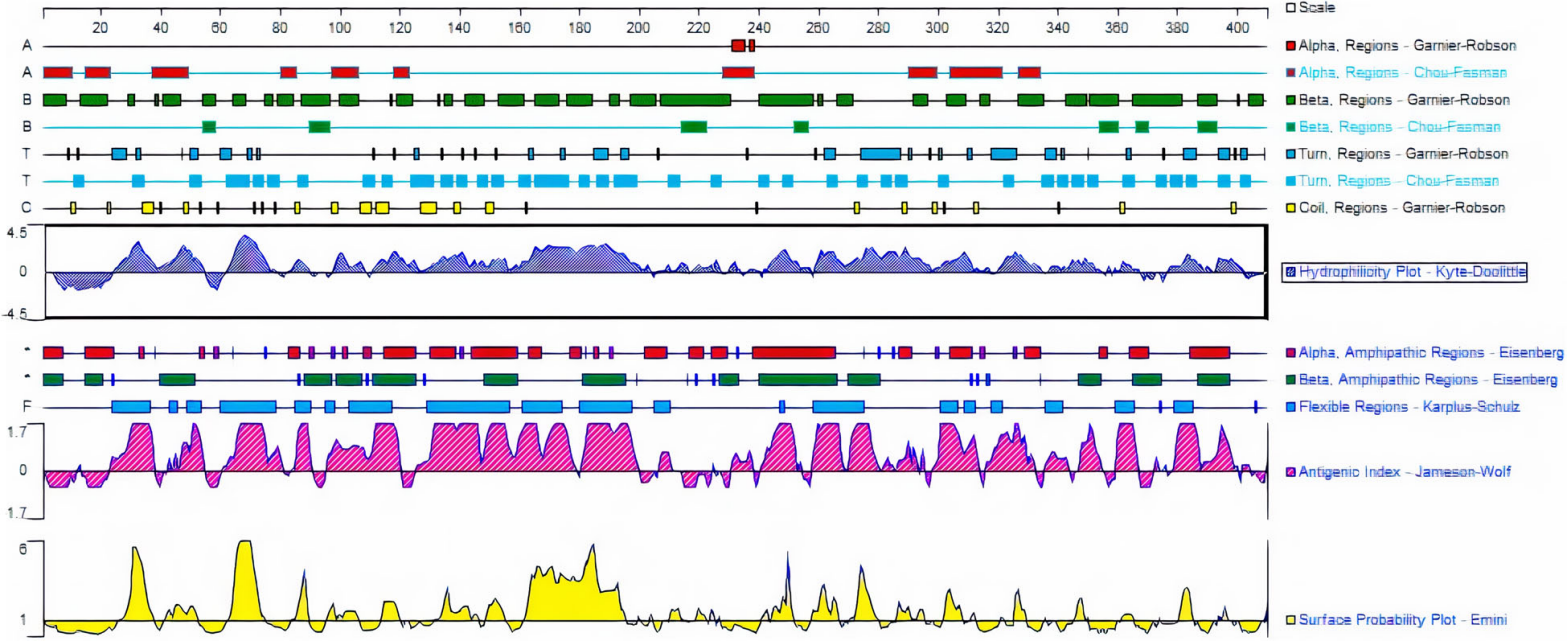
Protein structure prediction using DNASTAR. Multiple peaks in the antigenic index contributes to a potential antigenic determinant of lysyl oxidase protein

Using the PSIPRED online tool, the secondary protein structure of canine lysyl oxidase gene was determined. Eight percent of total amino acids contributed to helix, 73% to coils, and 18% to strands (Fig. 2). This shows that coil dominated among the secondary structure elements followed by alpha helix. The dominant coiled structural content might be due to the presence of proline amino acid (hydrophobic). Proline has a special property of disrupting structured secondary structure by creating kinks in the polypeptide chains, thus resulting in coiling. Solvent accessibility can further aid in providing useful insights about the sequence and structure relationship. A total of 53% were predicted as buried, 19% medium, and 25% were exposed; about 41% positions were predicted as disordered. Further, 38% of total amino acids were predicted as non-polar, 34% polar, 14% hydrophobic, and 14% contributed to aromatic plus cysteine (Fig. 3). Human and rat lysyl oxidase propeptide has been predicted to contain more than 80% disordered residues [32]. As predicted by DNASTAR, multiple peaks in the antigenic index contributes to a potential antigenic determinant of lysyl oxidase protein (Fig. 4).

### Tertiary structure prediction

**Fig. 5:**
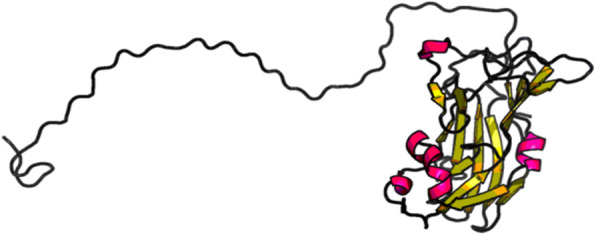
Homology model of canine lysyl oxidase protein by RAPTORX**.** Human LOX homolog 2 (5ze3.1.A) was used a modeling template with a significant similarity with the query sequence. The predicted oligo state of the protein model was monomer

**Fig. 6:**
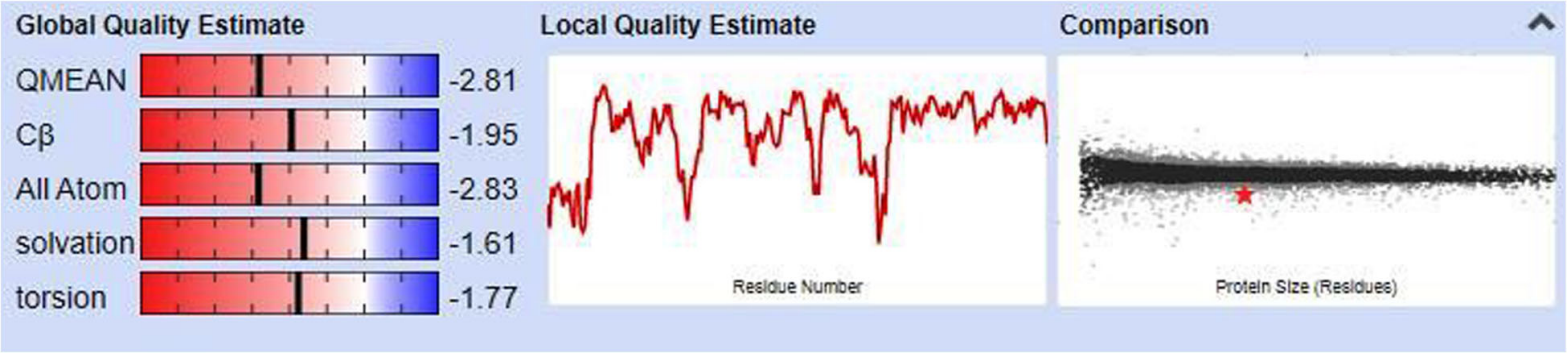
Evaluation of protein model. Based on QMEAN score and Z score, a good quality model was selected

**Fig. 7:**
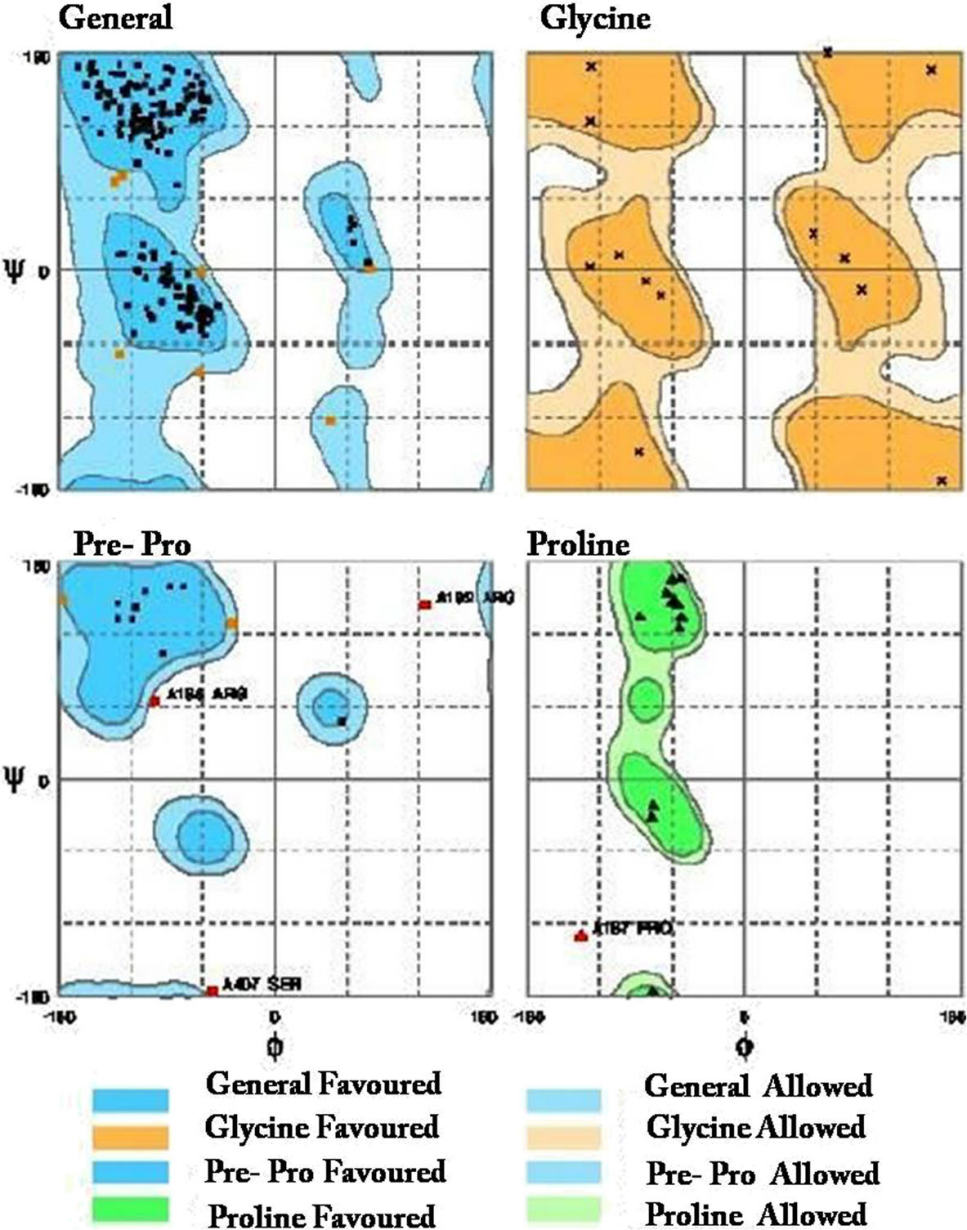
Structure Validation by Ramachandran plot depicts the general as well as specific distribution of amino acids. A total of 94.2% of amino acids were in favored and 2.23% were outliers suggesting higher degree of the stability of the predicted protein structure

Homology modeling was carried out to predict the 3-D structure of lysyl oxidase protein, since there is no experimental data available in the protein data bank. Lysyl oxidase 3-D structure model was generated by RaptorX online software and SWISS MODEL that works by selecting the best template for modeling (Fig. 5). Human LOX homolog 2 (5ze3.1.A) was used a modeling template with a significant similarity with the query sequence. The predicted oligo state of the protein model was monomer. Based on QMEAN score and Z score, a good quality model was selected (Fig. 6). The model was further validated by Ramachandran plot which concluded that 94.2% of amino acids were in favored and 2.23% were outliers (Fig. 7). More than 90% residues in favored region are attributes of a good quality model [33]. Similar type of in silico homology modeling has also been reported for human lysyl oxidase protein [34].

### Functional analysis

**Fig. 8:**
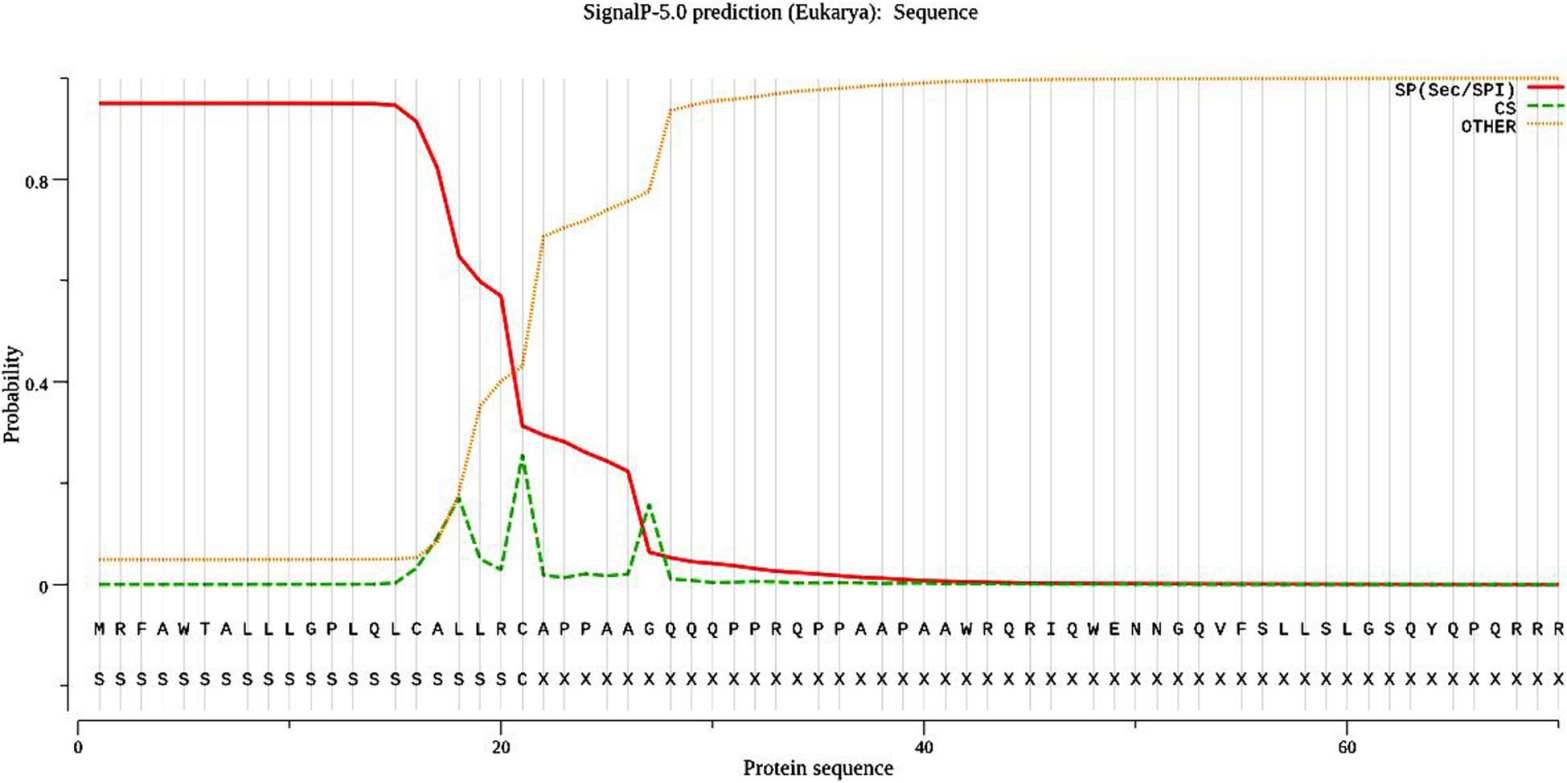
SignalP-5.0 prediction. Signal peptide likelihood was around 0.9505. Predicted location of peptide cleavage site was between position 21 and 22

A total of 12 cysteine residues at positions 16, 21, 230, 236, 283, 316, 322, 332, 343, 353, 390, 404 were found in the lysyl oxidase protein using the CYC_REC tool and forms at least 1 disulfide bridge. Cysteine residues are vital for protein’s thermostability while the disulfide bonds are important in folding of protein. Serine [10], threonine (5 ), and tyrosine [13] are predicted as potential phosphorylation sites. NetNGlyc online tool predicted two N-glycosylation sites at 96th and 136th position with high confidence. The SignalP 5.0 server predicts the incidence of signal peptides and the position of their cleavage sites. The likelihood of the signal peptide was around 0.9505 and the location of peptide cleavage site between position 21 and 22 was also predicted (Fig. 8). Through Psite software, it was found that the protein sequence has N-myristoylation site the maximum number of times. Also, a putative copper-binding region signature was predicted in the lysyl oxidase protein sequence at position 278-291. Copper atom exists within an octahedral coordination complex along with three histidine residues within the enzyme’s central region [35]. Other motif regions are summarized in (Table 3). ProtComp 9.0 revealed that the sub-cellular localization of the protein was extracellular (secreted). Functional analysis by InterproScan predicted protein’s biological role in oxidation-reduction process. Further, it has a copper ion binding and an oxidoreductase molecular activity.

**Table 3:** Motif regions present in the protein sequence

Motif information	No. of sites	Amino acid residues
N-glycosylation site.	2	96–99, 136–139
Protein kinase C phosphorylation site	1	303–305
Casein kinase II phosphorylation site	4	153–156, 289–292, 294–297, 320–323
Tyrosine kinase phosphorylation site	3	168–175, 168–175, 168–176
N-myristoylation site	6	52–57, 78–83, 107–112, 131–136, 338–343 342–347
Prenyl group-binding site	2	16–19, 404–407
Microbodies C-terminal targeting signal	3	153–155, 189–191, 294–296
Lysyl oxidase putative copper-binding region signature	1	278–291
Growth factor and cytokines receptors family signature 1	1	343–355

### Protein-protein interaction study

**Fig. 9:**
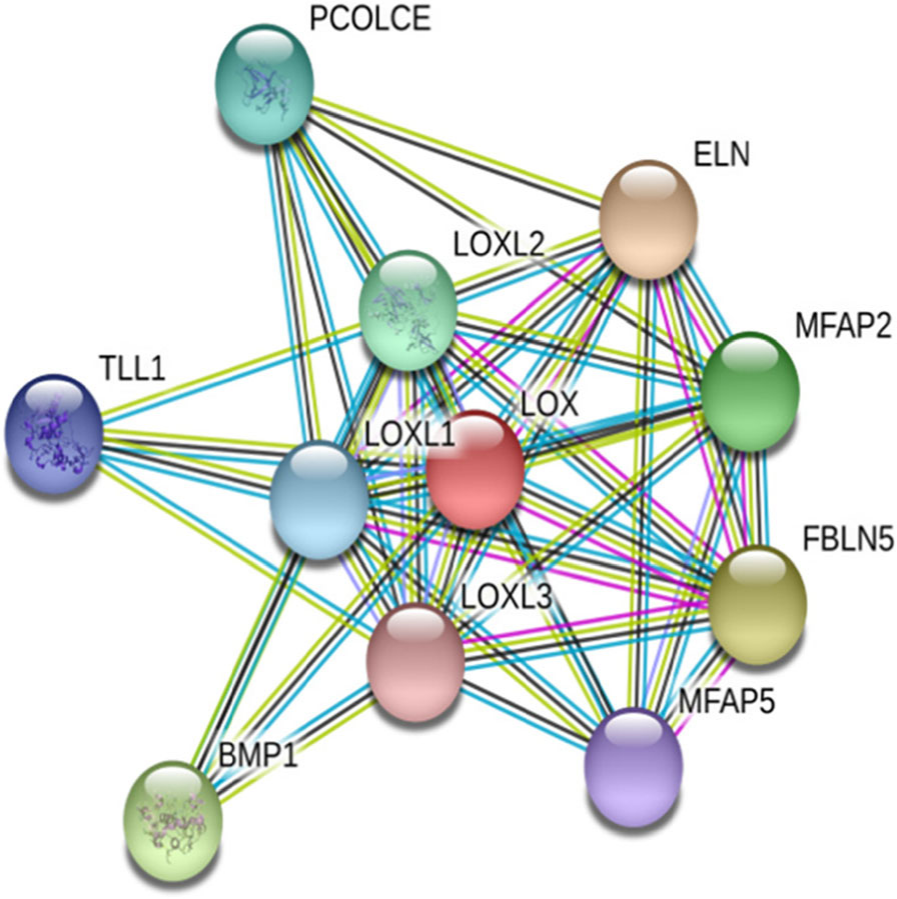
Protein-protein interaction map of canine lysyl oxidase protein by STRING web server. The closest interacting protein having the shortest node was found elastin while the distant interacting protein was lysyl oxidase-like 3 and microfibrillar-associated protein 5

**Fig. 10:**
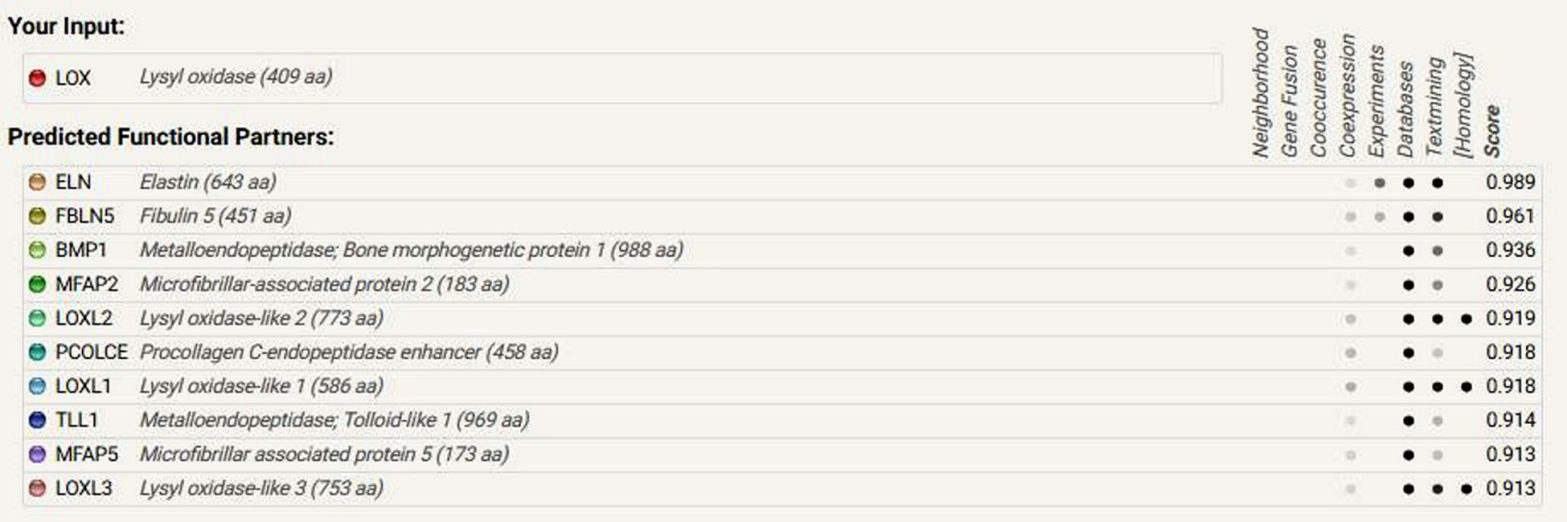
Screenshot from STRING server of predicting interacting proteins with the query sequence. Top 10 interacting partners have been displayed

Protein interaction network resolved by STRING web server revealed 10 potential interacting protein associates (Fig. 9) based on various network parameters like text mining, gene fusion, co-occurrence, co-expression, neighborhood, and databases. A node indicates a protein while as a connecting edge represents their interaction. The closest interacting protein having the shortest node was found elastin while the distant interacting protein was lysyl oxidase-like 3 and microfibrillar-associated protein 5. Potential interacting protein associates with canine lysyl oxidase protein are listed in (Fig. 10). Lysyl oxidase propeptide has been associated with interact with elastin, an extracellular protein promoting deposition onto elastic fibers [36]. Also, secreted LOX (proenzyme) is activated by bone morphogenetic protein 1 (BMP-1), releasing the mature catalytic domain and its N-terminal propeptide [37]. Proteins generally function by interacting with other proteins forming protein complexes and networks. Elucidating these complex protein interactions will give important clues as to the function of novel proteins that govern the cell behavior.

## Conclusion

Lysyl oxidase is a matrix remodeling enzyme which plays a vital role both inside and outside the cells contributing to cell matrix interactions, extracellular matrix assembly and organization. However, its aberrant expression (either upregulation or downregulation) in various pathological and physiological conditions is still being investigated. LOX is also being studied as a target for cancer metastasis owing to its incidence in various cancers. Thus, understanding the structural and functional properties of the protein will further facilitate a better understanding of its mechanism of enzyme action. Due to the non-availability of the crystal structure, studying the in silico structure-function aspects of the protein appears to be the moonlight in the dark. In this study, a flexible, unstable, hydrophilic, and extracellular protein with a molecular weight of 46 kDa was found. Functional motifs in the protein were also predicted along with a putative copper-binding region. Copper acts as a cofactor and a determinant of enzyme activity in the connective tissues. The predicted 3-D structure might help in shedding light on the biological functions. There is a likely prospect that the concerned gene in humans may have evolutionary relationship with that of canines and may be correlated with the cancer progression. The extracellular function of the canine LOX along with their elevated mRNA and protein expression in canine mammary tumors makes LOX a therapeutic target for diagnosis of mammary tumors. Targeting LOX in canine cancer is an exciting prospect for the development of drugs that could prevent cancer metastasis and progression. Thus, it becomes imperative to use bioinformatic tools to understand the relationship. This will help both veterinarians as well as medical experts in providing basic and concrete information regarding its diagnosis and treatment.

## Data Availability

All data generated or analyzed during this study are included in this published article.

## Abbreviations

LOX: Lysyl oxidase protein
*lox*: Lysyl oxidase gene
LTQ: Lysyl-tyrosylquinone
OHS: Occipital horn syndrome
BLAST: Basic Local Alignment Search Tool
MEGA: Molecular Evolutionary Genetic
ExPASy: Expert Protein Analysis System
SIB: Swiss Institute of Bioinformatics
UPGMA: Unweighted pair group method
Mw: Molecular weight
pI: Isoelectric point
EC: Extinction coefficients
II: Instability index
AI: Aliphatic index
GRAVY: Grand average hydropathicity
